# Optimal Branching Structure of Fluidic Networks with Permeable Walls

**DOI:** 10.1155/2017/5284816

**Published:** 2017-05-21

**Authors:** Vinicius R. Pepe, Luiz A. O. Rocha, Antonio F. Miguel

**Affiliations:** ^1^Department of Mechanical Engineering, Federal University of Rio Grande do Sul (UFRGS), Porto Alegre, RS, Brazil; ^2^Mechanical Engineering Graduate Program, University of Vale do Rio dos Sinos (UNISINOS), São Leopoldo, RS, Brazil; ^3^Department of Physics, School of Science and Technology, University of Evora, Evora, Portugal

## Abstract

Biological and engineering studies of Hess-Murray's law are focused on assemblies of tubes with impermeable walls. Blood vessels and airways have permeable walls to allow the exchange of fluid and other dissolved substances with tissues. Should Hess-Murray's law hold for bifurcating systems in which the walls of the vessels are permeable to fluid? This paper investigates the fluid flow in a porous-walled T-shaped assembly of vessels. Fluid flow in this branching flow structure is studied numerically to predict the configuration that provides greater access to the flow. Our findings indicate, among other results, that an asymmetric flow (i.e., breaking the symmetry of the flow distribution) may occur in this symmetrical dichotomous system. To derive expressions for the optimum branching sizes, the hydraulic resistance of the branched system is computed. Here we show the T-shaped assembly of vessels is only conforming to Hess-Murray's law optimum as long as they have impervious walls. Findings also indicate that the optimum relationship between the sizes of parent and daughter tubes depends on the wall permeability of the assembled tubes. Our results agree with analytical results obtained from a variety of sources and provide new insights into the dynamics within the assembly of vessels.

## 1. Introduction

In nature, the function of many flow systems is to deliver a fluid flow from a finite-size volume to one point (and vice versa) [1]. Tree-shaped networks provide the solution to house and facilitate fluid flow. They play a vital role in the organization and operation of tracheobronchial system, blood vessels, river basins, and so forth. These networks branch by dichotomy with a regular reduction of their length and diameter, and they have been found to have fractal properties [2–5]. The tip of each tube bifurcates to form two daughter tubes, along the length of the network. Repetition of these bifurcations generates the stereotyped, hierarchically organized branched architecture of the tree [6]. Therefore, bifurcation is the building block of trees and deserves to be analyzed.

The sizes of the tubes in the bifurcation are important factors in determining the efficiency of physiological processes. Despite the remarkable variety and complexity of natural tree-shaped flow networks, a relationship exists between parent and daughter tube sizes [1, 6–8]. The studies of Hess [9] and Murray [10] show that, in the cardiovascular system, when a parent vessel branches into daughter vessel, the cube of the parent vessel's diameter equals the sum of the cubes of the daughter vessels' diameters. This reduction of diameter of daughter vessels is essential for a proper functioning of the cardiovascular system and is usually termed as Hess-Murray law. This law predicts the relationship between the diameters of parent and daughter tubes for internal flows obeying laminar conditions. Hess-Murray's law has been most often applied to symmetry and asymmetric branching. In both cases it is assumed that the pressure drop over the daughter tubes is similar which is valid for a symmetry bifurcation but is only appropriated for a very low degree of branching asymmetry [11]. For a symmetric bifurcation, the ratio of daughter and parent tubes' diameters can be written more simply as equal to a homothetic factor of 2^-1/3^.

Hess-Murray law can be derived based on minimization of the energy required to synthesize, maintain, and pump blood (principle of minimum work) [10], minimization of flow resistance under the constraints posed by the space (constructal law) [1, 12], minimization of volume under constant pressure drop and flow rate [13], applying to a constant shear stress in all tubes [14], and so forth.

Using the constructal law, Bejan et al. [12] later derived an equation predicting the lengths of branching tubes by minimizing the overall flow resistance over a finite-size space. For laminar flow, they also found that the cube of the length of a parent tube should be equal to the sum of the cubes of the lengths of the daughter tubes. This means that in a symmetric bifurcation, for example, successive tubes are also homothetic with a size ratio of 2^-1/3^. Uylings [15] and Bejan et al. [12] derived equations predicting the sizes of branching tubes whose internal flows obey turbulent conditions. Revellin et al. [16] and Miguel [17] also presented extensions of Hess-Murray's law for non-Newtonian fluids that exhibit shear-thinning and shear-thickening behavior. Miguel [18] analyzes the optimal arrangement of vessels when there is dependence of apparent viscosity of blood on vessel diameter and hematocrit (Fåhræus-Lindqvist effect). Following the Haynes marginal zone theory, he obtain comprehensive expressions for the branching sizes of parent and daughter vessels providing easier flow access.

Although targeting the cardiovascular tree [9], experimental data have shown Hess-Murray's law holds well in medium sized blood vessels [17], in the respiratory tract airways of warm-blooded vertebrates such as humans and dogs [10], and in the tubes for fluid transport in plants [19]. This means the vascular and bronchial trees not only in warm-blooded vertebrates but also in tree flow systems of animals and plants have reached similar design solutions.

From the considerable amount of literature comparing Hess-Murray's law to physiological studies, there are also cases where vessels deviate from the optimum perspective [20–22]. Studies show deviations from the Hess-Murray law for proximal bifurcations of aorta and some pulmonary veins [20, 21]. The diameter of acinar airways (respiratory region of lungs) seems to fall less steeply than that of conducting airways that obey Hess-Murray law [22, 23]. Some authors point out that these deviations might be due to structural constraints. They argue that since deviations are small, the penalty of deviating from Hess-Murray's law is also small. Thus, structural constraints are likely to influence their design [21]. Miguel [23] derived analytical expressions for the optimum way to connect parent and daughter vessels together having permeable walls. This work has brought out the idea for the optimum branching sizes of vessels influenced by the wall permeability. Although important, the analytical approach presented in this paper is based on several assumptions [23] and is lacking 3D details that can enrich the understanding of the influence of the permeable boundary [24].

Generally, in the body, fluid flow is laminar and there are evidences that turbulent flows may even pose health risk [25, 26]. Notice also that tubes that are part of the circulatory and respiratory systems may be able to transport fluids and solutes across the walls. For example, a network of capillaries (vascular tree) surrounds the respiratory tree (acinus zone) and brings blood into close proximity with air within the alveolus [27]. The exchange of oxygen and carbon dioxide is accomplished through semipermeable walls of both alveoli and capillaries.

This paper seeks to answer the following question: Are small deviations from the Hess-Murray law observed in permeable vessels tolerated because of small punitive increase of resistance penalty, or is there optimum way to connect parent and daughter vessels together to achieve the higher performance? Here, we present a numerical study devised to investigate the influence of wall permeability on the optimum geometrical relationship governing the ratio of sizes of the tubes in a branching network. The study focuses specifically on T bifurcation transporting fluid under steady laminar flow of incompressible fluids. Analytical expression for the optimum daughter-parent sizes ratio is presented and compared with Hess-Murray law.

## 2. Methods

### 2.1. Geometrical Configuration of Branching Tubes in a T-Junction

**Figure 1:**
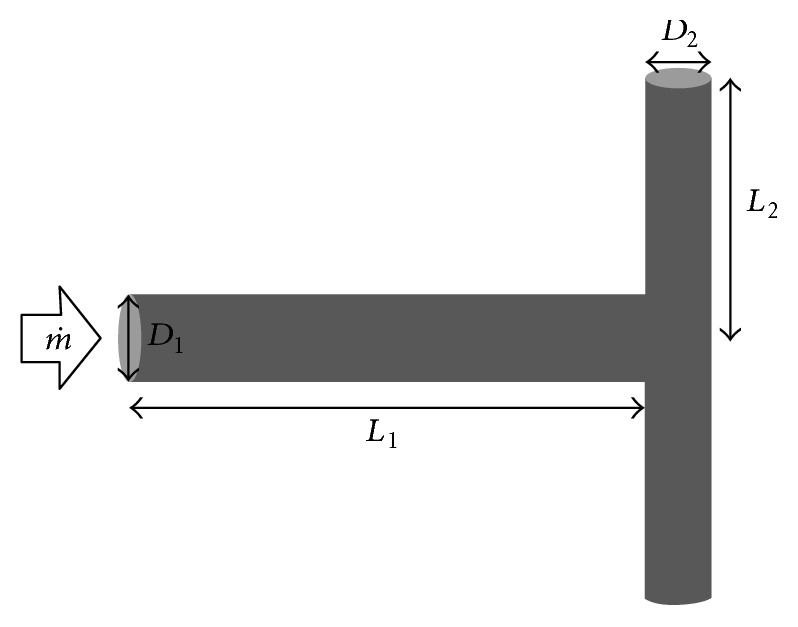
Schematic representation of a T-shaped flow structure ($\dot{m}$, mass flow rate,* D*, diameter, and* L*, length).

Figure 1 illustrates the symmetrical T-shaped assembly of cylindrical tubes. Parent tube bifurcates and its size may change by a certain factor. The ratio of successive diameters and lengths (daughters to parent vessels) can be written as

(1) D2D1=aD,L2L1=aL,

where *a*_*D*_ and *a*_*L*_ are scale factors or homothety ratios, *D* is the diameter, *L* is the length, and the subscripts 1 and 2 mean parent and daughter tubes. According to Hess-Murray law, for laminar flow, *a*_*D*_ = *a*_*L*_ = 2^-1/3^ but in this paper branching tubes with homothety ratios between 0.1 and 1.0 are also studied. In order to compare the performance of each assembly of tubes to fluid flow, the following constraints are considered:

(2) π4D12L1+2D22L2=const,2L1L2=const.

The constraints represented by these equations physically define that both the total volume occupied by the tubes and the total space occupied by the planar assembly of tubes are fixed, respectively.

The optimal branching rule for the assembly of tubes (best design) is the one that maximizes the flow access (or minimizes the total flow resistance) subject to constraints (see (2)).

### 2.2. Numerical Modeling

The standard finite volume method is used to solve the coupled Navier–Stokes equations under steady-state, isothermal, and incompressible conditions. Thus, the essence of our phenomenological description is the following set of equations:

(3) ∇v→=0,

(4) ρv→∇v→=−∇P+μ∇2v→,

where *v* is the velocity, *ρ* is the density, *μ* is the dynamic viscosity, and *P* is the pressure.

For a porous wall, flow is modeled by adding an extra source term to the momentum conservation equation (see (4)). Here, we assume that this source term *S* is due to viscous effects only (Darcy flow [28–30]):

(5) $$\begin{array}{c} \overset{\to }{S}=\frac{\mu }{K}\vec{v}, \end{array}$$

where* K* is the permeability of the porous material which measures the ability of a material to transmit fluid through itself [29, 30].

These governing equations were solved in Ansys FLUENT software [31] using the segregated method with implicit formulation. The COUPLED algorithm with underrelaxation was selected for the pressure-velocity coupling. For (4), the convective term is discretized using second-order-upwind scheme in order to obtain sufficiently accurate solutions. In order to obtain a stable and accurate iterative process, the relaxation factors for momentum and pressure were set to 0.75 and 0.75, respectively. The residual values of governing equations (3) and (4) were all set to 10^−4^ and 10^−6^, respectively.

At the parent tube, fluid is introduced at a constant mass flow rate and at a temperature of 309.15 K. The fluid is expelled to the outside through the daughter tubes. Nonslip boundary conditions are considered along solid obstacles-fluid interface, and the pressure exterior of the configuration is constant along the axial. In this study, laminar flows of two Newtonian fluids are studied. The Reynolds number, based on the hydraulic diameter of the inlet tube, used in all computations is 10^3^. Both air (*ρ* = 1.1405 kg/m^3^; *μ* = 1.9043 × 10^-5 ^Pa·s) and blood (*ρ* = 1060 kg/m^3^; *μ* = 2.78 × 10^-3 ^Pa·s) are used as fluids in the simulations. While air is considered a Newtonian fluid, blood is associated with non-Newtonian effects. However, in large arteries and veins, non-Newtonian effects cease because of high shear rates and blood behaves as a Newtonian fluid [32] which is the case reported here. The wall permeability of branching tubes is assumed to be constant along the assembly and ranging from 0 m^2^ (impermeable wall) to 10^-1 ^m^2^.

**Figure 2:**
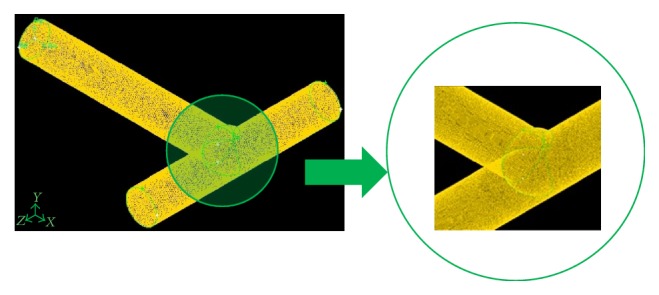
Grid of the domain for the T-shaped flow structure (*D*_2_*/D*_1_ =* L*_2_*/L*_1_ = 2^-1/3^).

Ansys GAMBIT software [33] was used to build the grid over the branching tubes geometries. A grid independence analysis was performed and various grid sizes were investigated. A computational grid containing around 1.6 million tetrahedral elements was found to be appropriate (Figure 2).

## 3. Optimum Branching Design for an Optimal Flow Access

According to Murray [10] an optimum arrangement of vessels for fluid flow is achieved with the least possible biological work. Although originally derived from the principle of minimum work, Hess-Murray law can be obtained based on other optimization principles [1, 8, 11–18].

The occurrence of flow configuration (design) is a universal phenomenon that occurs everywhere, whether in animate and inanimate systems, social dynamics (pedestrian traffic flows), or manmade systems. The constructal law [1] is about the generation of configuration throughout flow system. It resolves many contradictory ad hoc statements of “optimality,” end design, such as minimum and maximum statements (entropy, mass, power, etc.). This law states that for a finite-size flow system to persist in time its configuration must evolve in such a way that it provides easier access to its currents. Easier access means minimum flow resistance (viscous friction), because these thermodynamic “imperfections” cannot be avoided. Therefore, the optimal T-shaped flow structure is the one whose impact (penalty) on the access to fluid flow is minimum, which means that the total resistance is minimum.

The search for the branching design offering the minimum total resistance can be performed based on

(6) $$\begin{array}{c} R=\frac{\Delta P}{\phi }, \end{array}$$

where *R* is the total flow resistance and *ϕ* is the mass flow rate. The results can be also presented in a dimensionless form according to

(7) $$\begin{array}{c} R^{*}={\frac{R}{R}}_{a_{D}=a_{L}=2^{-1/3}}. \end{array}$$

Here *R*^*∗*^ is the total dimensionless flow resistance and *R*_*a*_*D*_=*a*_*L*_=2^−1/3^_ is the flow resistance in a T-shaped assembly of vessels designed according to *D*_2_/*D*_1_ = *L*_2_/*L*_1_ = 2^−1/3^.

## 4. Results

The velocity and pressure fields in the T-shaped structures were numerically obtained for a Reynolds number of 1000.

**Figure 3:**
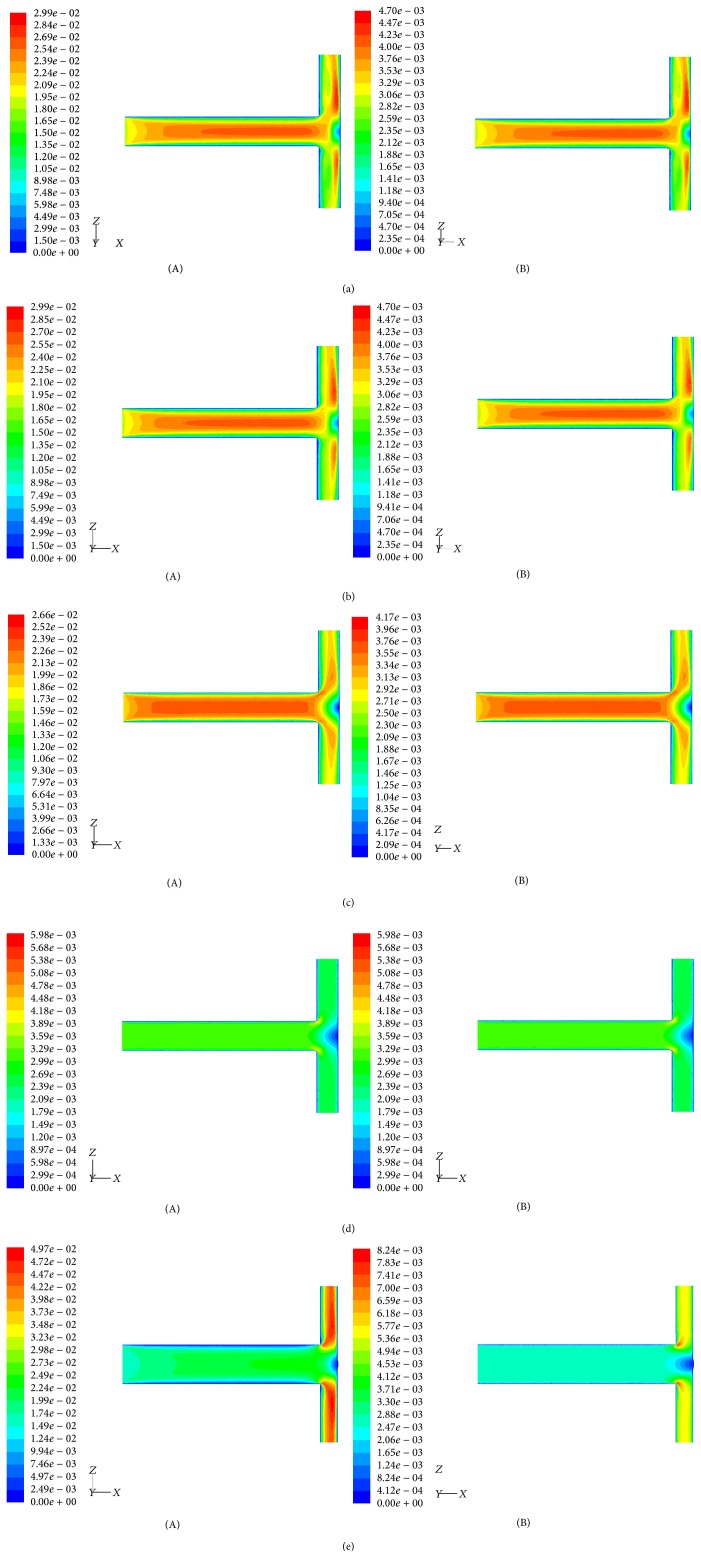
(a) Velocity contours (middle plane) for a T-shaped structure designed according to* D*_2_*/D*_1_ =* L*_2_*/L*_1_= 2^-1/3^ and impervious walls (*K* = 0 m^2^): (A) air (*ρ* = 1.1405 kg/m^3^; *μ* = 1.9043 × 10^−5^ Pa·s); (B) blood (*ρ* = 1060 kg/m^3^; *μ* = 2.78 × 10^−3^ Pa·s). (b) Velocity contours (middle plane) for a T-shaped structure designed according to* D*_2_*/D*_1_ =* L*_2_*/L*_1_ = 2^-1/3^ and permeable walls (*K* = 10^−5^ m^2^): (A) air (*ρ* = 1.1405 kg/m^3^; *μ* = 1.9043 × 10^−5^ Pa·s); (B) blood (*ρ* = 1060 kg/m^3^; *μ* = 2.78 × 10^−3 ^Pa·s). (c) Velocity contours (middle plane) for a T-shaped structure designed according to* D*_2_*/D*_1_ =* L*_2_*/L*_1_ = 2^-1/3^ and permeable walls (*K* = 10^−3 ^m^2^): (A) air (*ρ* = 1.1405 kg/m^3^; *μ* = 1.9043 × 10^−5^ Pa·s); (B) blood (*ρ* = 1060 kg/m^3^; *μ* = 2.78 × 10^−3^ Pa·s). (d) Velocity contours (middle plane) for a T-shaped structure designed according to* D*_2_*/D*_1_ =* L*_2_*/L*_1_ = 2^-1/3^ and permeable walls (*K* = 10^−1^ m^2^): (A) air (*ρ* = 1.1405 kg/m^3^; *μ* = 1.9043 × 10^−5^ Pa·s); (B) blood (*ρ* = 1060 kg/m^3^; *μ* = 2.78 × 10^−3^ Pa·s). (e) Velocity contours (middle plane) for a T-shaped structure designed according to* D*_2_*/D*_1_ = 0.5 and* L*_2_*/L*_1_ = 2^-1/3^ air (*ρ* = 1.1405 kg/m^3^; *μ* = 1.9043 × 10^−5^ Pa·s): (A) impervious walls (*K* = 0 m^2^); (B) permeable walls (*K* = 10^−1^ m^2^).

**Figure 4:**
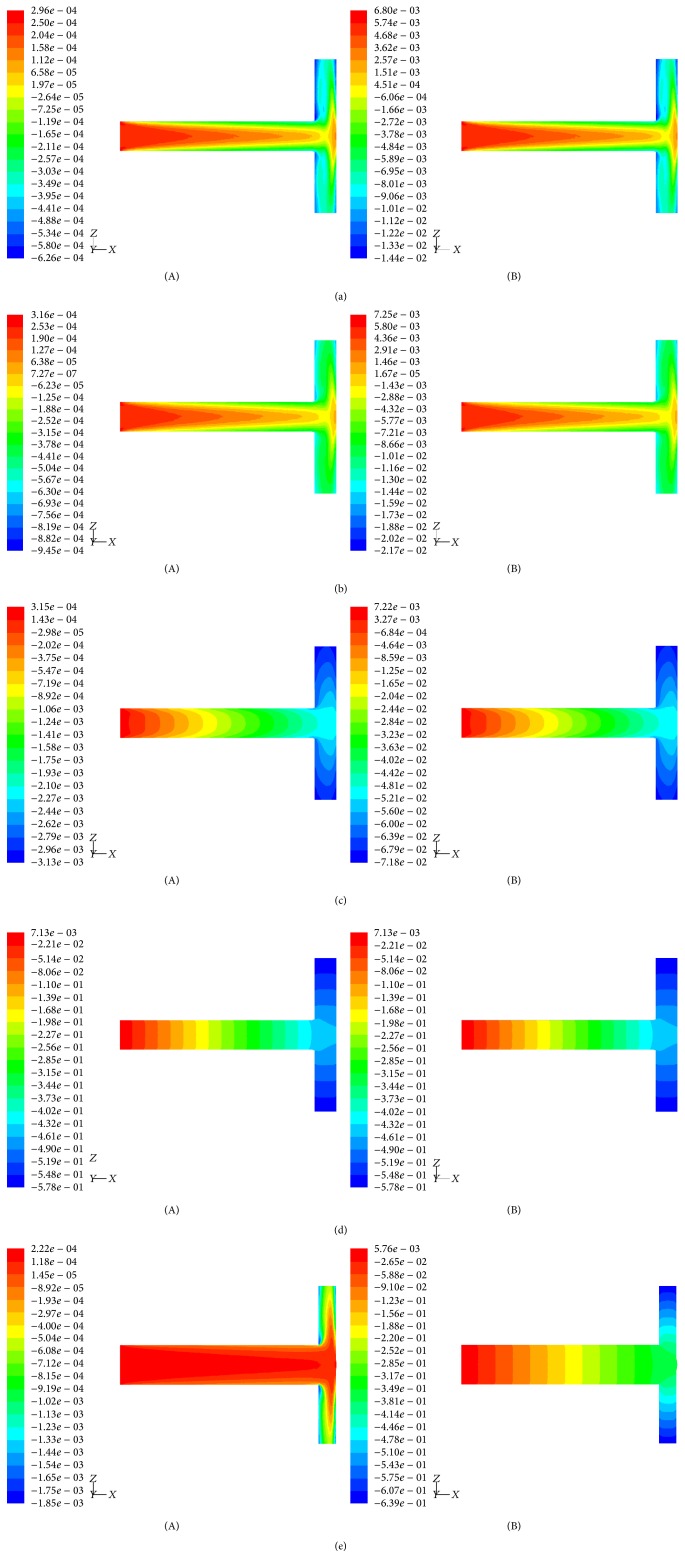
(a) Contours of total pressure (middle plane) for a T-shaped structure designed according to* D*_2_*/D*_1_ =* L*_2_*/L*_1_ = 2^-1/3^ and impervious walls (*K* = 0 m^2^): (A) air (*ρ* = 1.1405 kg/m^3^; *μ* = 1.9043 × 10^−5^ Pa·s); (B) blood (*ρ* = 1060 kg/m^3^; *μ* = 2.78 × 10^−3 ^Pa·s). (b) Contours of total pressure (middle plane) for a T-shaped structure designed according to* D*_2_*/D*_1_ =* L*_2_*/L*_1_ = 2^-1/3^ and permeable walls (*K* = 10^−5^ m^2^): (A) air (*ρ* = 1.1405 kg/m^3^; *μ* = 1.9043 × 10^−5^ Pa·s); (B) blood (*ρ* = 1060 kg/m^3^; *μ* = 2.78 × 10^−3^ Pa·s). (c) Contours of total pressure (middle plane) for a T-shaped structure designed according to* D*_2_*/D*_1_ =* L*_2_*/L*_1_ = 2^-1/3^ and permeable walls (*K* = 10^−3^ m^2^): (A) air (*ρ* = 1.1405 kg/m^3^; *μ* = 1.9043 × 10^−5^ Pa·s); (B) blood (*ρ* = 1060 kg/m^3^; *μ* = 2.78 × 10^−3 ^Pa·s). (d) Contours of total pressure (middle plane) for a T-shaped structure designed according to* D*_2_*/D*_1_ =* L*_2_*/L*_1_ = 2^-1/3^ and permeable walls (*K* = 10^−1 ^m^2^): (A) air (*ρ* = 1.1405 kg/m^3^; *μ* = 1.9043 × 10^−5 ^Pa·s); (B) blood (*ρ* = 1060 kg/m^3^; *μ* = 2.78 × 10^−3 ^Pa·s). (e) Contours of total pressure (middle plane) for a T-shaped structure designed according to* D*_2_*/D*_1_ = 0.5 and* L*_2_*/L*_1_ = 2^-1/3^ air (*ρ* = 1.1405 kg/m^3^; *μ* = 1.9043 × 10^−5 ^Pa·s): (A) impervious walls (*K* = 0 m^2^); (B) permeable walls (*K* = 10^−1 ^m^2^).

Velocity and total pressure contours taken for air and blood flow thorough T-shaped geometries with impermeable wall (*K* = 0 m^2^) and with permeability between 10^−5^ and 10^-1 ^m^2^ are depicted in Figures 3 and 4.

**Figure 5:**
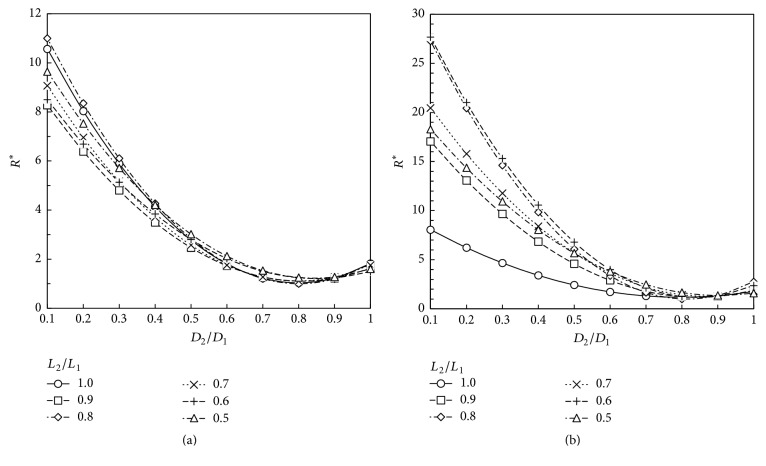
Dimensionless flow resistance, *R*^*∗*^, for a T-shaped structure with impervious walls (*K* = 0 m^2^): (a) air (*ρ* = 1.1405 kg/m^3^; *μ* = 1.9043 × 10^−5 ^Pa·s); (b) blood (*ρ* = 1060 kg/m^3^; *μ* = 2.78 × 10^−3 ^Pa·s).

**Figure 6:**
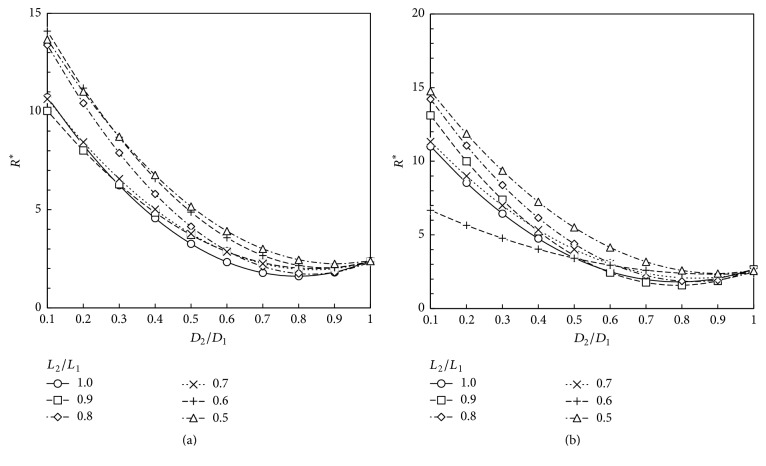
Dimensionless flow resistance, *R*^*∗*^, for a T-shaped structure with permeable walls (*K* = 10^−5 ^m^2^): (a) air (*ρ* = 1.1405 kg/m^3^; *μ* = 1.9043 × 10^−5 ^Pa·s); (b) blood (*ρ* = 1060 kg/m^3^; *μ* = 2.78 × 10^−3 ^Pa·s).

**Figure 7:**
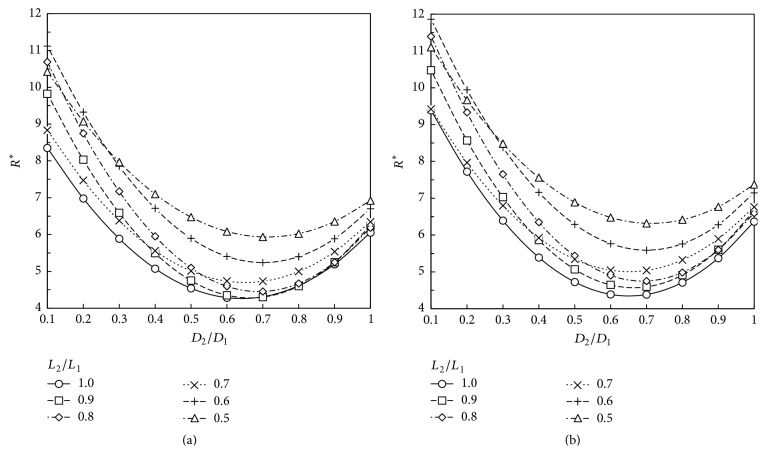
Dimensionless flow resistance, *R*^*∗*^, for a T-shaped structure with permeable walls (*K* = 10^−3 ^m^2^): (a) air (*ρ* = 1.1405 kg/m^3^; *μ* = 1.9043 × 10^−5 ^Pa·s); (b) blood (*ρ* = 1060 kg/m^3^; *μ* = 2.78 × 10^−3 ^Pa·s).

**Figure 8:**
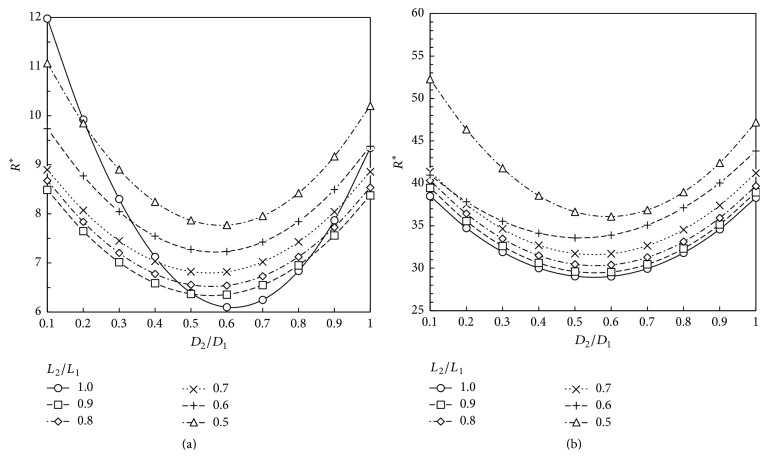
Dimensionless flow resistance, *R*^*∗*^, for a T-shaped structure with permeable walls (*K* = 10^−1 ^m^2^): (a) air (*ρ* = 1.1405 kg/m^3^; *μ* = 1.9043 × 10^−5 ^Pa·s); (b) blood (*ρ* = 1060 kg/m^3^; *μ* = 2.78 × 10^−3 ^Pa·s).

 Figures 3(a)–3(d) and 4(a)–4(d) show that the velocity and total pressure contours are similar for both air and blood flows. This means that Newtonian fluids under the same Reynolds number exhibit similar behavior. Our simulations (Figures 3(a)(A) and 3(e)(A) and Figures 4(a)(A) and 4(e)(A)) also indicate that velocity and total pressure contours are highly sensitive to the homothety ratios *a*_*D*_ and *a*_*L*_. T-shaped assembly of vessels designed with optimal ratios (see Figures 5–8) show a more uniform distribution. Moreover, the results depicted at Figures 3(a)–3(e) and 4(a)–4(e) show a strong influence of wall permeability on both velocity and total pressure contours. Note that the presence of holes along the walls leads to fluid flow through the walls, which significantly affects the flow dynamics as well as the stress distribution, regardless of fluid properties.

It is remarkable to notice that an asymmetric flow distribution occurs in symmetric T-shaped assembly of vessels (Figures 3(a)–3(d)). This inhomogeneity of the flow distribution is the result of the effect of inertia on the momentum transport, and the Reynolds number should significantly influence it. This agrees with the 2D study of a symmetric Y-assembly of rectangular channels performed by Andrade Jr. et al. [34]. Geometrical constraints also influence the inhomogeneity of the flow distribution. According to Figures 3(a) and 3(e) we conclude that the asymmetry flow is less significant for a lower homothety ratio between diameters of daughter and parent tubes* a*_*D*_. This means that the area available when fluid flow meets the daughter vessels influences the flow symmetry. Figures 3(a)–3(d) also underline the progressive influence of the wall permeability on the flow distribution. A strong decrease of flow asymmetry with the permeability is observed.

In summary, the asymmetric flow occurring in symmetric T-shaped geometries is determined by not only the Reynolds number and the homothety ratio* a*_*D*_, but also by the ability of the vessel wall to transmit fluid through itself.

Another interesting result is that, at the point where fluid flow meets the bifurcation, as the flow negotiates the sharp corners a small zone of flow separation is visible. At opposite side of wall, flow also becomes detached to form a separation zone with a free boundary layer. This region is characterized by lower shear stresses than the opposite side of daughter wall. As evident from Figures 3(a)–3(e), the region of flow separation is not dependent on fluid properties (viscosity and density) but varies markedly with both the wall permeability and the homothety ratios (*a*_*D*_,* a*_*L*_). It is possible to associate regions of more pronounced flow separation with branched structures designed with nonoptimal ratios (see Figures 5–8). On the other hand, flow separation becomes negligible for higher permeabilities.

From Figures 3(a)–3(e) and 4(a)–4(e), there are some conclusions to be drawn. First, the optimum way to connect parent and daughter vessels together is not dependent on the fluid properties. Second, the existence of low shear separation zones, where fluid has smaller velocity, and regions of higher shear, with different velocities and pressure magnitudes and in different sites of the daughter tubes, which depends on the wall permeability and on the vessel sizes, contributes to different resistance of the T-assembly of tubes. An implication of these results is noteworthy. The optimum arrangement of vessels is dependent on both size of vessels and wall permeability. For this reason, to obtain the optimal solution for the vessels arrangement, we study next the dependence of flow resistance on* a*_*D*_,* a*_*L*_, and* K*.

Based on our numerical results, the total dimensionless flow resistance defined according to (7) is obtained for the geometries studied. These results are depicted at Figures 5–8. These plots show that the dimensionless flow resistance is a function concave up in the interval 0 <* a*_*D*_ ≤ 1, with a minimum resistance at the bottom of the curve.

Figures 5–8 show dependence of flow resistances on the fluid properties, homothety ratios (*a*_*D*_,* a*_*L*_), and wall permeabilities. As expected from the analyses of Figures 3(a)–3(e) and 4(a)–4(e), one observes that the shift of minimum resistance with *a*_*D*_ is only dependent on the wall permeability. As mentioned before, Miguel [23] presented an analytical approach for fluid flow through porous-walled tree-shaped networks. This approach considers that the losses on the connection of large and small vessels together are negligible compared with friction losses through the vessels. The junction losses have a sizeable effect on optimized geometry when a dimensionless parameter called svelteness, defined by the ratio between the external and internal length scales, is lower than the square root of 10 [35]. Therefore, our results cannot be directly compared to analytic study of [23] since the svelteness of our T-assembly of tubes ranges from 6.4 to 7.2. However, general trends of variation can be compared. As noted in Miguel's study, the total flow resistance depends on the homothety ratio of the branching vessels as well as on the wall permeability of vessels, which agree with our numerical results. Our results also match the findings obtained by Bejan et al. [12] for T-assemblies of tubes with impervious walls.

According to the constructal law [1, 7, 12], maximum flow access means minimum flow resistance subjected to the space constraints represented by (2). Thus, the optimum way to connect parent and daughter tubes together to achieve the fastest access to fluid flow can be obtained from Figures 5–8.  Table 1 shows the optimum homothety ratio* a*_*D*_ when the tube segments are part of a configuration obeying* a*_*L*_ = 2^-1/3^. This is particularly suitable for network of tubes that must fit inside of space with a characteristic length constraint: the reduction of tube length by a constant factor occurs in both the respiratory and cardiovascular trees. Optimality may be also sought for both diameters and lengths.  Table 2 shows the optimum homothety ratios *a*_*D*_ and *a*_*L*_ with the wall permeability.

As expected from the analyses of Figures 3(a)–3(e) and 4(a)–4(e), Tables 1 and 2 show that the optimum homothety ratios* a*_*D*_ and* a*_*L*_ are independent of the fluid properties (viscosity and density). For tubes with impervious walls, both homothety ratios for diameters and lengths must obey a scale factor of 0.8 which agrees with Hess-Murray law and the findings of Bejan et al. [12] (i.e.,* a*_*D*_ =* a*_*L*_ = 2^-1/3^). In addition, our results agree with the findings of Miguel [23] indicating that the optimum homothety ratios *a*_*D*_ and *a*_*L*_ depend on the permeability of vessel walls.

A comparison between Tables 1 and 2 shows that the optimum homothety ratio* a*_*D*_ is the same for an assembly of tubes obeying* a*_*L*_ = 2^-1/3^ or for T configuration of tubes with optimum lengths and diameters. This is a remarkable result that testifies the robustness of this optimum* a*_*D*_ result.

## 5. Conclusion

Hess-Murray law is attempted to explain the best way to connect bifurcating tubes by predicting the relationship between the diameters of parent and daughter tubes at each bifurcation. In this paper, we attempt to describe the best design of a T-shaped assembly of tubes with permeable walls. A numerical study of Newtonian fluids under laminar flow conditions is carried out in assembly of tubes with different scale factors or homothety ratios for diameters and lengths. In order to compare the performance of each assembly of tubes to fluid flow, both the total volume occupied by the tubes and the total space occupied by the planar structure are fixed.

Among other results, we show that an asymmetric flow occurs in symmetric T-branched structures. Inertial force seems to break the symmetry of the flow distribution and, as a result, it depends on Reynolds number. The flow asymmetry is also found to depend both on the wall permeability and on the homothety ratio* a*_*D*_. Our results show that T-assemblies of tubes are not homothetic with a unique size ratio but present different values that depend on wall permeability. For diameters, the homothety ratio decreases with wall permeability but the homothety factor for lengths increases with the wall permeability. Different homothetic ratios are a necessary consequence to connect large and small vessels together to achieve maximum fluid-flow access. Successive tubes segments in the T configuration are homothetic with a size ratio of 2^−1/3^ (Hess-Murray law) only for tubes with impermeable walls.

## Figures and Tables

**Table 1:** Optimal branching diameters for the assembly of tubes with *L*_2_*/L*_1_ = 2^-1/3^.

*K* (m^2^)	Blood		Air	
	(*ρ* = 1060 kg/m^3^; *μ* = 2.78 × 10^−3^ Pa·s)		(*ρ* = 1.1405 kg/m^3^; *μ* = 1.9043 × 10^−5^ Pa·s)	
	(*D*_2_/*D*_1_)_optimum_	*R* ^*∗*^_minimum_	(*D*_2_/*D*_1_)_optimum_	*R* ^*∗*^_minimum_
0	0.8	1.0	0.8	1.0
10^−5^	0.8	1.8	0.8	1.7
10^−3^	0.7	4.8	0.7	4.5
10^−1^	0.6	30.6	0.6	6.5

**Table 2:** Optimal branching diameters and lengths for a T-shaped assembly of tubes.

*K* (m^2^)	Blood (*ρ* = 1060 kg/m^3^; *μ* = 2.78 × 10^−3^ Pa·s)			Air (*ρ* = 1.1405 kg/m^3^; *μ* = 1.9043 × 10^−5^ Pa·s)		
	(*D*_2_/*D*_1_)_optimum_	(*L*_2_/*L*_1_)_optimum_	*R* ^*∗*^_minimum_	(*D*_2_/*D*_1_)_optimum_	(*L*_2_/*L*_1_)_optimum_	*R* ^*∗*^_minimum_
0	0.8	0.8	1.0	0.8	0.8	1.0
10^−5^	0.8	0.8	1.6	0.8	0.8	1.6
10^−3^	0.7	0.9	4.4	0.7	0.9	4.3
10^−1^	0.6	0.9	29.3	0.6	1.0	6.1
